# Function discovery of a non-ribosomal peptide synthetase-like encoding gene in the nematode-trapping fungus *Arthrobotrys oligospora*

**DOI:** 10.3389/fmicb.2023.1210288

**Published:** 2023-07-13

**Authors:** Tiantian Gu, Hengqian Lu, Huiwen Liu, Guanghui Zhang, Yongzhong Wang

**Affiliations:** ^1^School of Life Sciences, Anhui University, Hefei, Anhui, China; ^2^Key Laboratory of Human Microenvironment and Precision Medicine of Anhui Higher Education Institutes, Anhui University, Hefei, Anhui, China; ^3^Anhui Key Laboratory of Modern Biomanufacturing, Hefei, Anhui, China

**Keywords:** nematode-trapping fungi, *Arthrobotrys oligospora*, NRPS-like, metabolomics, gene knock-out

## Abstract

In this study, the function of a non-ribosomal peptide synthetase-like (NRPS-like) encoding gene *AOL_s00188g306* (*g306*) was investigated to reveal the association between NRPS and nematocidal activity in the nematode-trapping fungus *Arthrobotrys oligospora*. Sequence analysis indicated that the encoded product of *g306* is an adenylation domain of non-ribosomal peptide synthetases and extended short-chain dehydrogenase/reductase domain-containing proteins, and displays a wide substrate spectrum. The Δ*g306* mutants were more sensitive to chemical stressors than the wild type. Disruption of *g306* impeded the nematocidal efficiency of *A. oligospora*. Metabolomics analysis showed that secondary metabolite biosynthesis and lipid metabolism were altered in the mutants. The phenotypic changes in the mutants can be attributed to the down-regulation of various metabolites, including fatty acyls, prenol lipids, steroidsand steroid derivative, and amino acid derivatives, identified in the present study. This study investigated the association between the non-ribosomal polypeptide-encoding gene *g306* and nematicidal activity in *A. oligospora*, providing a reference for resolving the predation mechanism of nematode-trapping fungus.

## 1. Introduction

Plant-parasitic nematodes are widespread globally and can cause various crop diseases (Xu et al., 2015), leading to yearly economic losses of over US$100 billion (Degenkolb and Vilcinskas, 2016). Therefore, controlling crop diseases caused by nematodes is essential. Chemically synthesized pesticides have been used to kill plant-parasitic nematodes (Pires et al., 2022). Fungal plant-parasitic nematodes can invade the roots of plants and enter host tissues, thereby limiting the effectiveness of chemical pesticides in controlling nematodes. Moreover, the potential toxicity of chemical pesticides poses a substantial threat to human health and environmental safety (Hussain et al., 2020). Thus, there is an urgent need for an environmentally friendly and effective method to prevent and control nematodes.

Nematophagous fungi are an important group of soil microorganisms that are widespread in aquatic and terrestrial ecosystems, and include over 200 species. These fungi represent a special class of microbial predators that can infect and kill harmful nematodes through various modes of action (Zhen et al., 2018). Based on their specific mode of action, nematophagous fungi can be classified as nematode-trapping (NT), endoparasitic, egg-parasitic, or toxin-producing fungi (Su et al., 2017). NT fungi can form different traps (such as adhesive networks, adhesive knobs, and constricting rings) to capture nematodes. The formation of traps is closely related to the efficiency of nematode predation by NT fungi (Xie et al., 2020). The mechanisms through which NT fungi infect nematodes and their ability to develop specialized mycelial traps to infect and consume nematodes has attracted considerable interest. However, the fungal infection of nematodes has been assumed to rely on more than trap formation alone.

NT fungi can produce several small-molecule metabolites that play an important role in nematode predation. Kuo et al. performed a comprehensive metabolomics study on 100 wild isolates of three NT fungal species. Molecular networking analysis revealed that they were capable of producing thousands of metabolites, and the chemical diversity of metabolites increased as the fungi transitioned to the predation phase. Moreover structural annotations revealed that these fungal metabolites belonged to various structural families, such as peptides, siderophores, fatty alcohols, and fatty acid amides (Kuo et al., 2020). *Arthrobotrys oligospora* is a representative of NT fungi and has promising applications in biological control as natural enemies of parasitic nematodes. Whole-genome sequencing indicated that *A. oligospora* has a strong ability to biosynthesize various secondary metabolites (Yang et al., 2011). The polyketide synthetase (PKSs), non-ribosomal peptide synthetases (NRPSs), and P450 families of enzymes are commonly involved in the synthesis of these secondary metabolites in fungi (Theobald et al., 2019; Mnguni et al., 2020). The PKS gene, *AOL_s00215g283*, in *A. oligospora* is involved in the production and formation of many secondary metabolites and fungal traps (Xu et al., 2016). Some *A. oligospora* nematocidal compounds, such as linoleic acid, the first nematocidal compound isolated from *A. oligospora*, and its levels positively correlated with the number of predatory traps formed by NT fungi in the predation phase (Stadler et al., 1993). *A. oligospora* can also produce volatile metabolites, such as furanone and pyrone, which are associated with transition from the saprophytic to pathogenic stages (Wang et al., 2018). Moreover, 6-methyl salicylic acid, produced by *Duddingtonia flagrans*, was confirmed to lure nematodes and modulate predation trap formation (Yu et al., 2021). 6-methylsalicylic acid and *m*-cresol also showed significant nematocidal activity against southern root-knot nematodes (Xu et al., 2016). Therefore, small-molecule metabolites play a critical role in NT fungi nematocidal abilities.

*A. oligospora* is a typical NT fungal species that has been extensively studied as a model for nematode-trapping fungi. As natural enemies of parasitic nematodes, they have promising applications for biological control. The non-ribosomal polypeptide synthase can synthesize a variety of secondary metabolites, however, there is a lack of research on the role of these substances in the nematode predation activity of *A. oligospora* so far. In this study, we investigated the NRPS-like encoding gene *g306* in *A. oligospora* using sequence analysis, gene-knockout experiments, and non-targeted metabolomic analysis. The sequence characteristics of *g306* were revealed, and the association between *g306* and *A. oligospora* nematocidal activity was investigated using metabolomic analysis. Our results suggest that *g306* plays an important role in regulating trap formation and NT fungi nematocidal abilities by influencing the biosynthesis of various small molecular metabolites.

## 2. Materials and methods

### 2.1. Strains and culture conditions

*A. oligospora* ATCC 24927 was purchased from the American Type Culture Collection (ATCC, Manassas, VA, USA) and preserved at the Anhui Provincial Human Microecology and Precision Medicine Laboratory. *A. oligospora* was cultured on potato dextrose agar plates (PDA, potato 200 g/L, glucose 20 g/L, and agar 15 g/L; natural pH) at 28°C. The pUC19 and pUC57 plasmids were stored in *Escherichia coli* DH5α and cultured in Luria broth (LB) medium (tryptone Caenorhabditis 10 g/L, yeast extract 5 g/L, sodium chloride 10 g/L, and agar 15 g/L) at 37°C. *Caenorhabditis elegans*, which was stored in our laboratory, was cultured in nematode growth medium (NGM) (3 g/L NaCl, 2.5 g/L peptone, 1.5 g/L agar, 5 mg/L cholesterol-ethanol solution, 1 mM MgSO_4_, 1 mM CaCl_2_, and 12.5 mM KH_2_PO_4_-K_2_HPO_4_) at 20°C.

### 2.2. Multiple sequence alignment and homologous structural domain analysis

Nucleotide and amino acid sequences of gene *g306* (Gene ID: 22897766) were obtained from the National Center for Biotechnology Information (NCBI), and conserved structure analysis and sequence alignment were performed using Basic Local Alignment Search Tool (BLAST). The core enzymes, structural domains, and products were predicted using the anti-SMASH database and Minimum Information about a Biosynthetic Gene cluster (MIBiG) (Kautsar et al., 2020; Blin et al., 2021).

### 2.3. Construction of knockout vectors

*AOL_s00188g306* of *A. oligospora* was knocked out using protoplast transformation based on homologous recombination (Si et al., 2022). The hygromycin-resistant gene (*hygR*) was selected as the screening marker gene, and knockout plasmids with the *hygR* screening marker gene were constructed using the pEASY-Uni-seamless Cloning and Assembly kit (Transgen Biotech, Beijing, China).

The genomic DNA of *A. oligospora* was extracted using a Fungal Genome Extraction Kit (Solarbio, Beijing, China). The upstream and downstream homologous arm sequences of *g306* were amplified using primers *g306*-3F/*g306*-3R and *g306*-5F/*g306*-5R (Supplementary Table S1) with genomic DNA as a template. The *hygR* fragment was amplified from the pUC57*-HygR* plasmid using the primers *Hph*-F/*Hph*-F (Supplementary Table S1). The pUC19 plasmid was double digested using restriction enzymes XbaI and BamHI (Transgen Biotech, Beijing, China), and the three fragments and linearized pUC19 plasmid were linked using a pEASY-Uni-seamless Cloning and Assembly kit (Transgen Biotech). The ligation product was transformed into *E. coli* DH5α, and the recombinant plasmid (pUC19-*g306*KO-*hygR*) was screened on LB plates containing ampicillin and verified *via* sequencing.

### 2.4. Protoplast transformation and screening of positive transformants

The wild-type (WT) strain of *A. oligospora* was cultured on PDA in a constant temperature incubator at 28°C for 7 days and inoculated into TG liquid medium (1% trypsin and 1% glucose). After incubation at 28°C for 16 h, it was further cultivated at 28°C for 24 h at 160 rpm in a constant-temperature shaking incubator. At the end of incubation, the mycelia were collected by filtration using a sterile funnel (with four layers of built-in sterile filter paper). The collected mycelium was rinsed using MN buffer (0.3 M MgSO_4_, 0.3 M NaCl), suspended in a mixture of cellulase and snailase prepared using the MN buffer (2,3 snailase solution:cellulase solution), and subjected to enzymatic digestion at 30°C with constant shaking at 160 rpm for 4 h. After enzymatic hydrolysis, the enzymatic solution was filtered, and the protoplasmic precipitate was collected by centrifugation at 3000 × *g* for 10 min; the protoplasmic precipitate was washed using KTC buffer (1.2 M KCl, 10 mM Tris–HCl, and 50 mM CaCl_2_) and suspended to obtain the protoplasmic suspension.

Using the knockout plasmid (pUC19-*g306*KO-*hygR*) as a template, the full-length sequences of the three fragments (5′-flanking, 3′-flanking, and thaumatin resistance gene fragments) were amplified using PCR with primers *g306*-3F and *g306*-3F (Supplementary Table S1). Protoplast suspension (100 μL) was mixed well with 10 μg of the knockout fragment PCR product, placed in an ice bath for 40 min, 700 μL of KTC buffer was added, mixed well, and incubated at 28°C for 1 h. Subsequently, the above suspension was spread on a TB3 plate (200 g/L sucrose, 3 g/L tryptone, 3 g/L yeast extract, and 0.75% agar), which was incubated at a constant temperature of 28°C for 18 h to allow regeneration. The TB3 plate was covered with a layer of TB3 containing 150 μg/mL of thaumycin B medium as the upper plate for transformer screening and incubated at 28°C for approximately 7 days to allow transformants to grow (Dong et al., 2021).

The transformants grown on TB3 plates were transferred to TYGA plates (10 g/L tryptone, 10 g/L glucose, 5 g/L yeast extracts, 5 g/L molasses, 1.5% agar powder) and incubated at 28°C for 5 days. The mycelium was collected and ground in liquid nitrogen, and the genomic DNA of the transformants was extracted using a fungal genome extraction kit. Positive transformants were screened using the validation primers YZ-F/YZ-R (Supplementary Table S1) and via PCR using genomic DNA as the template (Dong et al., 2021).

### 2.5. Determination of strain growth and stress resistance

To investigate the differences in growth rate and resistance between WT and knockout strains, *A. oligospora* WT and knockout strains (Δ*g306*) were cultured on PDA medium at 28°C for 6 days. Subsequently, circular mycelial discs (0.9 mm in diameter) of the same size as the WT and knockout strains, after 6 days of culture, were inoculated on 9 cm plates with different media. The growth status and growth rate of the knockout and WT strains on PDA, CMA, and TYGA media were compared. To test for the differences in resistance between knockout and WT strains, their growth rates were compared on TG plates containing 5–15 mM H_2_O_2_, 0.1–0.3 M NaCl, and 0.02–0.04% SDS (Zhang et al., 2022). The growth diameter and morphology of the colonies were recorded every 24 h (Peng et al., 2022; Si et al., 2022). The experiment was repeated three times.

### 2.6. Determination of conidia numbers and germination rate

To study the sporulation of WT and knockout strains, *A. oligospora* WT and gene-knockout strains (Δ*g306*) were grown on PDA medium and cultivated at 28°C for 6 days. Subsequently, circular mycelial discs (0.9 mm in diameter) of the WT and knockout strains, after 6 days of culture, were inoculated on a 9 cm plate of CMA medium. After incubation on CMA plates at 28°C for 10 days, the mycelium was rinsed well using sterile water and removed by filtration through a sterile funnel; the spore suspension was collected for conidia counting using a hemocytometer plate. Spore suspensions, diluted to the same concentration, were applied to 35 mm water agar plates, the number of spores germinated was observed microscopically every hour, and the spore germination rates of WT and knockout strains were counted at different time intervals (Peng et al., 2022; Si et al., 2022). The experiment was repeated three times.

### 2.7. Predation trap formation and nematode predatory efficiency

To compare the differences in predation trap formation and nematode predation between the WT and knockout strains, conidial suspensions of both strains were coated on 35 mm water agar plates and incubated at 28°C for 3 days. The nematodes were rinsed using M9 Buffer (5 g/L NaCl, 3 g/L KH_2_PO_4_, 6 g/L Na_2_HPO_4_ and add 1 mL of 1 M MgSO_4_ after cooling) to obtain a nematode suspension, and the average number of nematode strains in 1 μL of suspension was counted by microscopy. One thousand nematodes were added to the water agar plates and induced to form predatory traps. The number of predation traps formed and the nematode capture rates were counted at 6, 24, and 36 h after the addition of nematodes (Peng et al., 2022; Si et al., 2022). The experiment was repeated three times in triplicate.

### 2.8. Metabolomics analysis

The *A. oligospora* WT and gene knockout strain (Δ*g306*) were simultaneously cultured on TYGA plates at a constant temperature of 28°C for 1 week, and the mycelia were collected from the plates as a pre-sample. The sample (25 mg) was then weighed. After the addition of 500 μL of extract solution (methanol–water, 3:1 (v/v), with isotopically labeled internal standard mixture), the samples were vortexed for 30 s, sonicated for 10 min in an ice-water bath, and incubated for 1 h at −40°C to precipitate proteins. The sample was subsequently centrifuged at 12,000 *× g*, for 15 min at 4°C. The resulting supernatant was transferred into a fresh glass vial for analysis (Lu et al., 2019). A quality control sample was prepared by mixing an equal aliquot of the supernatants from all samples. *Liquid* chromatography mass spectrometry (LC–MS/MS) analysis was performed using a UHPLC system (Vanquish, Thermo Fisher Scientific) with a UPLC HSS T3 column (2.1 mm × 100 mm, 1.8 μm) coupled to a Q Exactive HFX mass spectrometer (Orbitrap MS, Thermo). The A phase used was aqueous, containing 5 mmol/L ammonium acetate and 5 mmol/L acetic acid, and the B phase contained acetonitrile. The sample tray temperature was 4°C, and the injection volume was 2 μL. The QE HFX mass spectrometer was used because of its ability to acquire MS/MS spectra using the information-dependent acquisition mode using the acquisition software (Xcalibur, Thermo). In this mode, the acquisition software continuously evaluated the full-scan MS spectrum. The ESI source conditions were set as follows: sheath gas flow rate, 30 Arb; Aux gas flow rate, 25 Arb; capillary temperature, 350°C; full MS resolution, 60,000; MS/MS resolution, 7,500; collision energy, 10/30/60 in NCE mode; and spray voltage, 3.6 kV (positive) or − 3.2 kV (negative). Raw data were converted to the mzXML format using ProteoWizard and processed using an in-house program, which was developed using R based on XCMS, for peak detection, extraction, alignment, and integration (Huan et al., 2017). Subsequently, an in-house MS2 database was used for metabolite annotation. The cut-off for annotation was set at 0.3.

### 2.9. Biostatistical analysis

Statistical analysis was performed using GraphPad Prism version 8.4.2 (GraphPad; San Diego, CA, USA). Differences at *p* < 0.05 were considered statistically significant.

## 3. Results

### 3.1. Sequencing analysis

**Figure 1:**
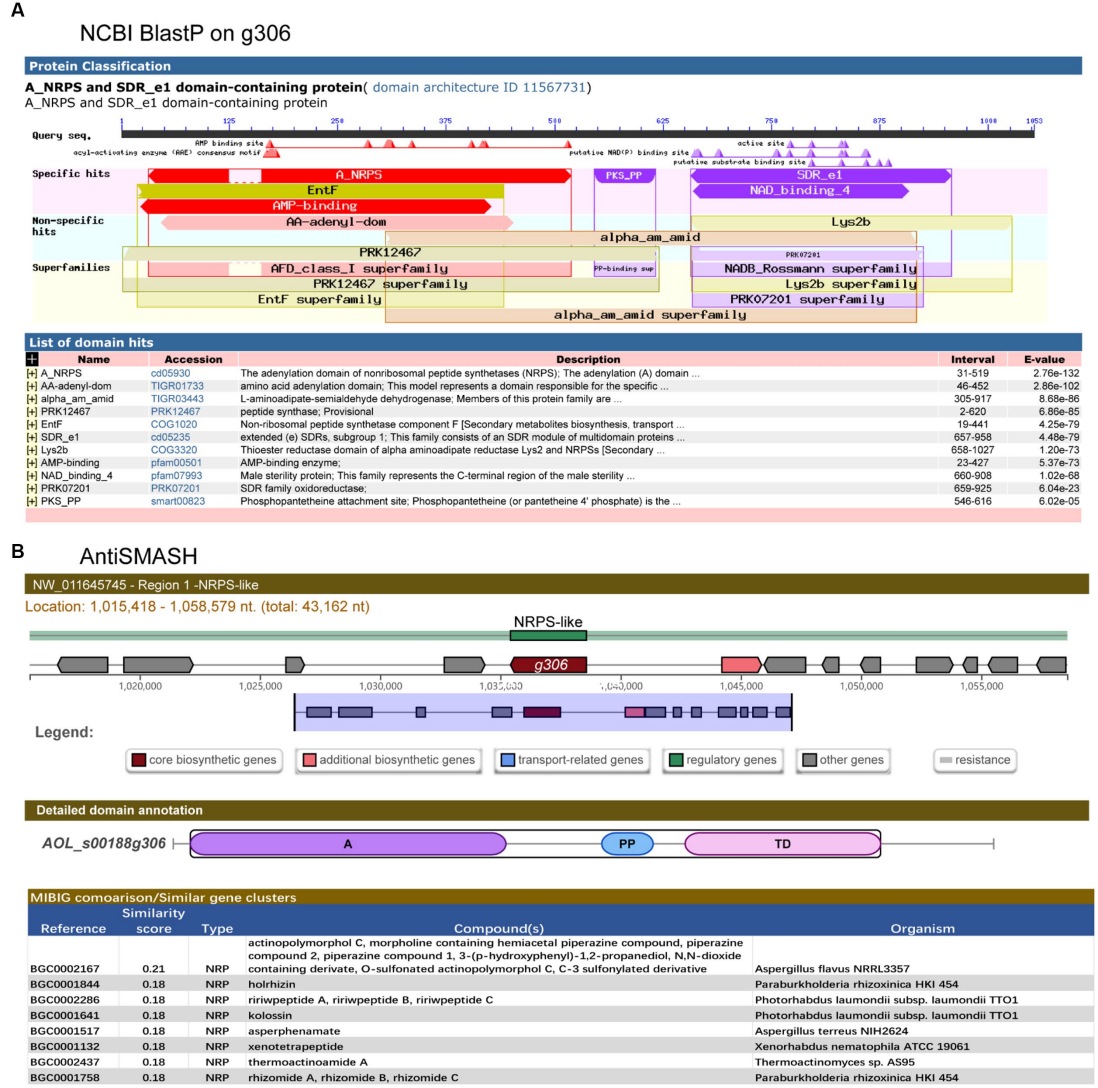
Structural domain analysis and secondary metabolite biosynthesis gene clusters prediction of *g306*. **(A)** The conserved domains of the *g306*-protein, as analyzed by NCBI Conserved Domain Search. **(B)** Gene *g306* was predicted as a core gene of NRPS-like biosynthesis gene clusters using AntiSMASH analysis. A: AMP-binding, PP: Phosphopantetheine attachment site, TD: thioester reductase structural domain.

A search of the NCBI database showed that the *g306* gene is 3,162 bp in length and encodes 1,053 amino acids. The encoded product of *g306* was annotated as an aminoacyl-ACP. Furthermore, the BlastP results indicated that the product of *g306* was classified as an adenylation domain of NRPSs (A_NRPS,cd05930) (aa31-519) and extended short-chain dehydrogenases/reductases (SDR_e1, cd05235) domain-containing protein (Figure 1A), which consists of an SDR module of multidomain proteins identified as putative PKSs, FASs, and NRPSs, among others. It catalyzes a wide range of activities, including the metabolism of steroids, cofactors, carbohydrates, lipids, aromatic compounds, and amino acids, and acts in redox sensing.

AntiSMASH predicted that *g306* is a core enzyme gene of the NRPS-like biosynthetic gene cluster, containing an adenylation domain (A), a phosphopantetheine attachment site (PP), and a thioester reductase domain (TD). We compared the non-ribosomal peptide synthesis gene clusters with previously reported. Some of the most similar products, such as piperazine analogs, were identified (Figure 1B).

Sequence analysis revealed that *g306* is a multidomain protein with a wide substrate spectrum.

### 3.2. *g306* knockout reduced stress resistance in *Arthrobotrys oligospora*

**Figure 2:**
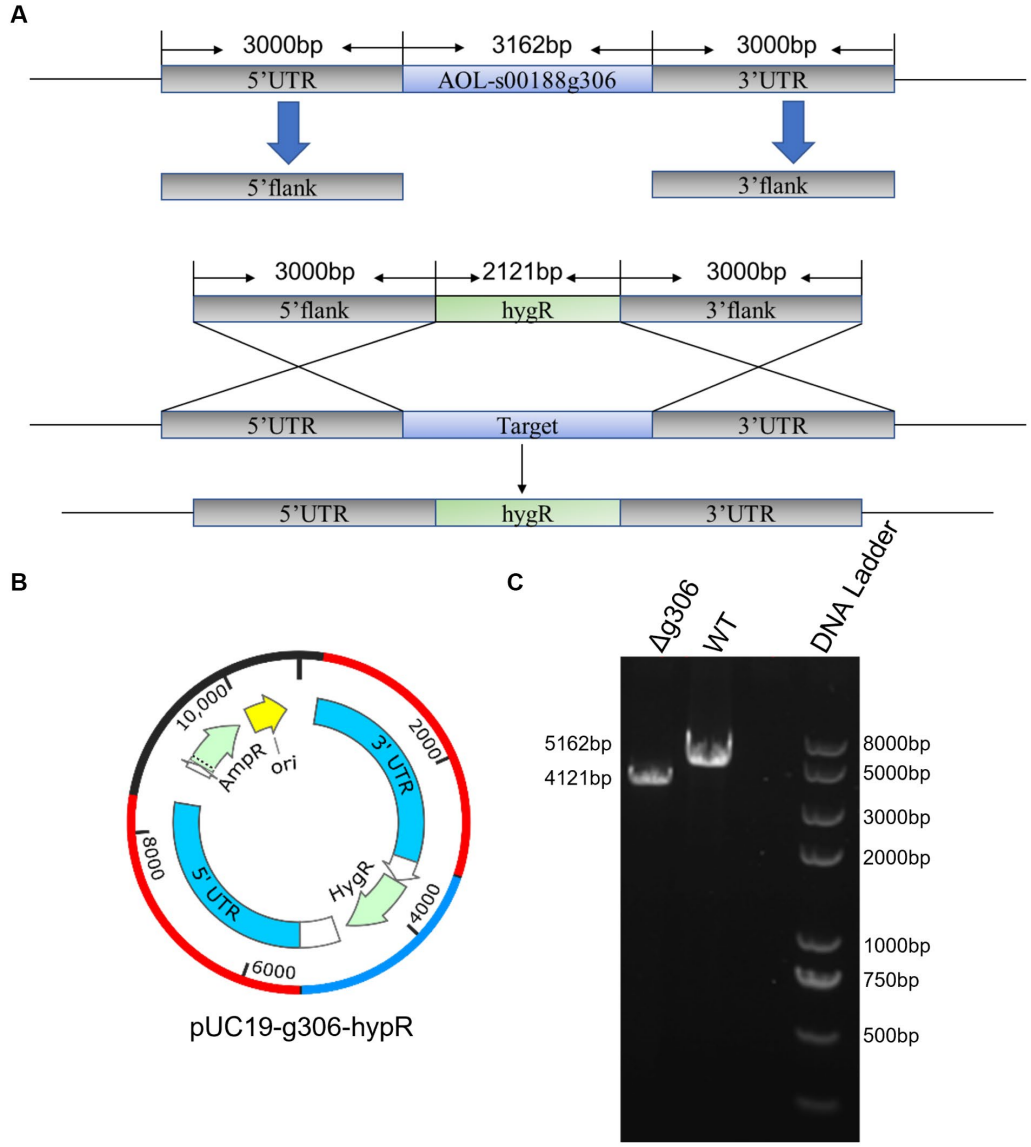
Construction of the *AOL-s00188g306* knockout strain. **(A)** Schematic diagram of gene knockout. **(B)** Construction of gene-knockout vector. **(C)** PCR verification of gene-knockout strains.

*g306* was knocked out by protoplast transformation using the principle of homologous recombination (Figure 2A). The knockout plasmids were mapped as in Figure 2B, and g306 knockout strain was screened as in Figure 2C, the WT strain amplicon was 5,162 bp, whereas the mutant strain was 4,121 bp. We screened a 4,121 bp amplification fragment from the 29 transformants using PCR, indicating that the *g306* knockout strains were successfully constructed.

**Figure 3:**
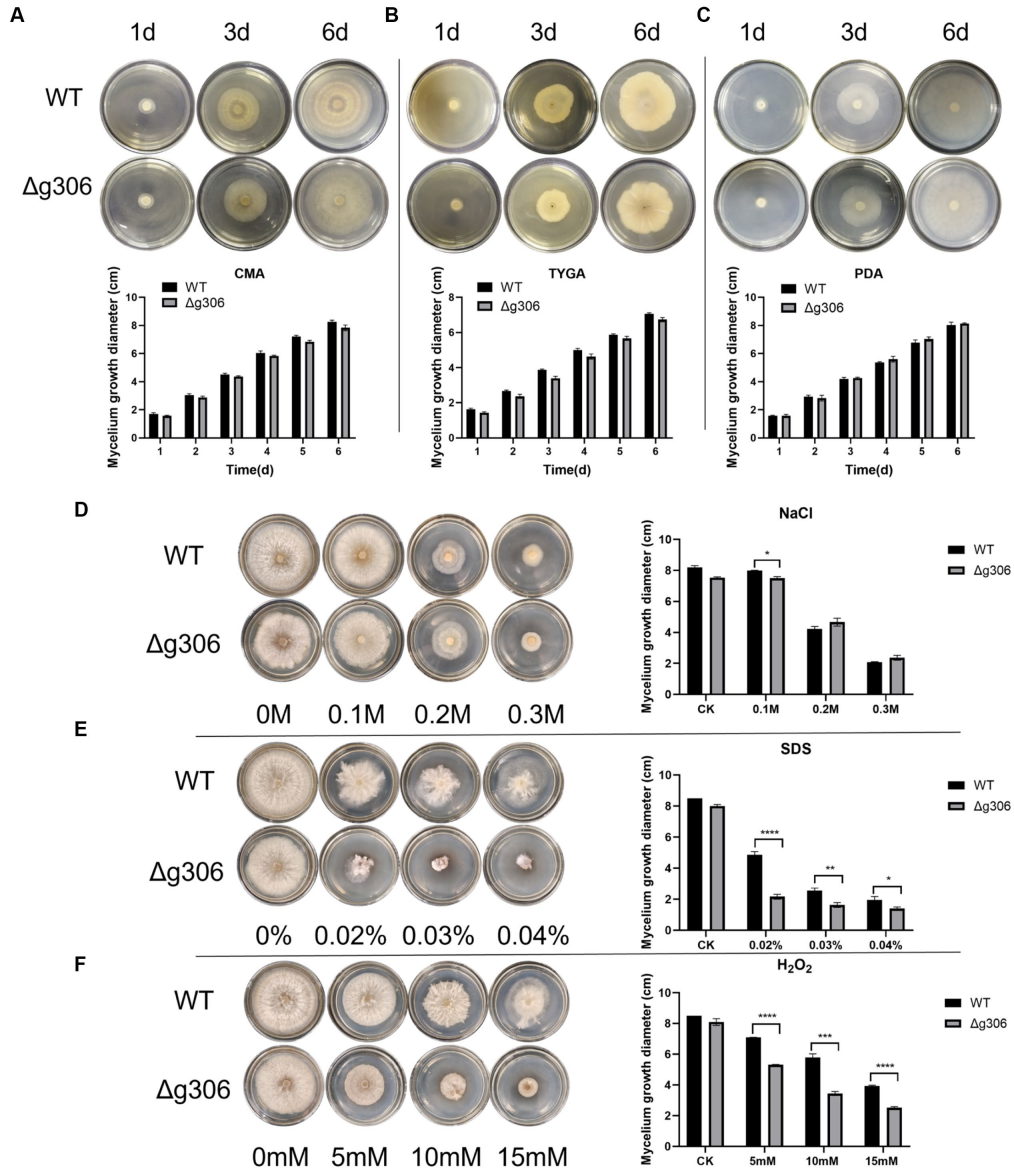
Effects of *g306* disruption on strains growth and stress resistance of *Arthrobotrys oligospora*. **(A–C)** Growth rates of *A. oligospora* WT and Δ*g306* mutants cultivated on CMA, TYGA and PDA plates for 6 days. **(B–F)** Growth of wild-type (WT) and Δ*g306* mutants on TG medium supplemented with different chemical stressors, including NaCl (0.1–0.3 M), SDS (0.02–0.04%) and H_2_O_2_ (5–15 mM).

The WT and Δ*g306* mutants were inoculated on PDA, CMA, and TYGA media to investigate the effects of *g306* knockout on *A. oligospora* growth. The growth diameters of the WT strain on CMA, TYGA and PDA plates after 6 days were 8.3, 7.1, and 8.0 cm, respectively (Figures 3A–C). The growth diameters of the Δ*g306* strain on CMA, TYGA, and PDA plates for up to 6 days were 7.8, 6.8, and 8.1 cm, respectively. Throughout the culture process, the growth status of the Δ*g306* strain was nearly identical to that of the WT strains on the three different media (Figures 3A–C).

To investigate the role of *g306* in the stress response, WT and Δ*g306* strains were compared for their responses to three chemical stressors, namely, oxidant H_2_O_2_, osmotic pressure regulator NaCl, and SDS-perturbed cell wall synthesis. The average diameter of the WT strain on 0.1 M NaCl plates was 8.2 cm. The growth rate of the Δ*g306* strain was slower (*p* < 0.05) than that of the WT strain, with an average diameter of 7.5 cm. The growth rate on 0.2 M and 0.3 M NaCl plates was nearly the same, with the average diameter of the WT strain on both plates being 4.3 and 2.1 cm, respectively, and the average diameter of the Δ*g306* strain on both plates being 4.7 and 2.3 cm, respectively. On plates containing different H_2_O_2_ and SDS concentrations, compared to the WT strain, the Δ*g306* mutants showed a significant reduction in growth rate (*p* < 0.05) (Figures 3D–F). By day 6, the average mycelial growth diameters of the WT strain on 0.02, 0.03, and 0.04% SDS plates were 4.9, 2.6, and 1.9 cm, respectively, whereas the average mycelial growth diameters of the Δ*g306* strain were 2.2, 1.6, and 1.4 cm, respectively, which were significantly lower than those of the WT strain (*p* < 0.05). On plates containing 5, 10, and 15 mM H_2_O_2_, the mycelial growth diameters of the WT strain were 7.1, 5.8, and 3.9 cm respectively, whereas the mycelial growth diameters of the Δ*g306* strain were 5.3, 3.5, and 2.5 cm, respectively. Thus, *g306* knockout significantly reduced the antioxidant capacity of the strain (*p <* 0.05).

### 3.3. *g306* knockout increased *Arthrobotrys oligospora* spore production

**Figure 4:**
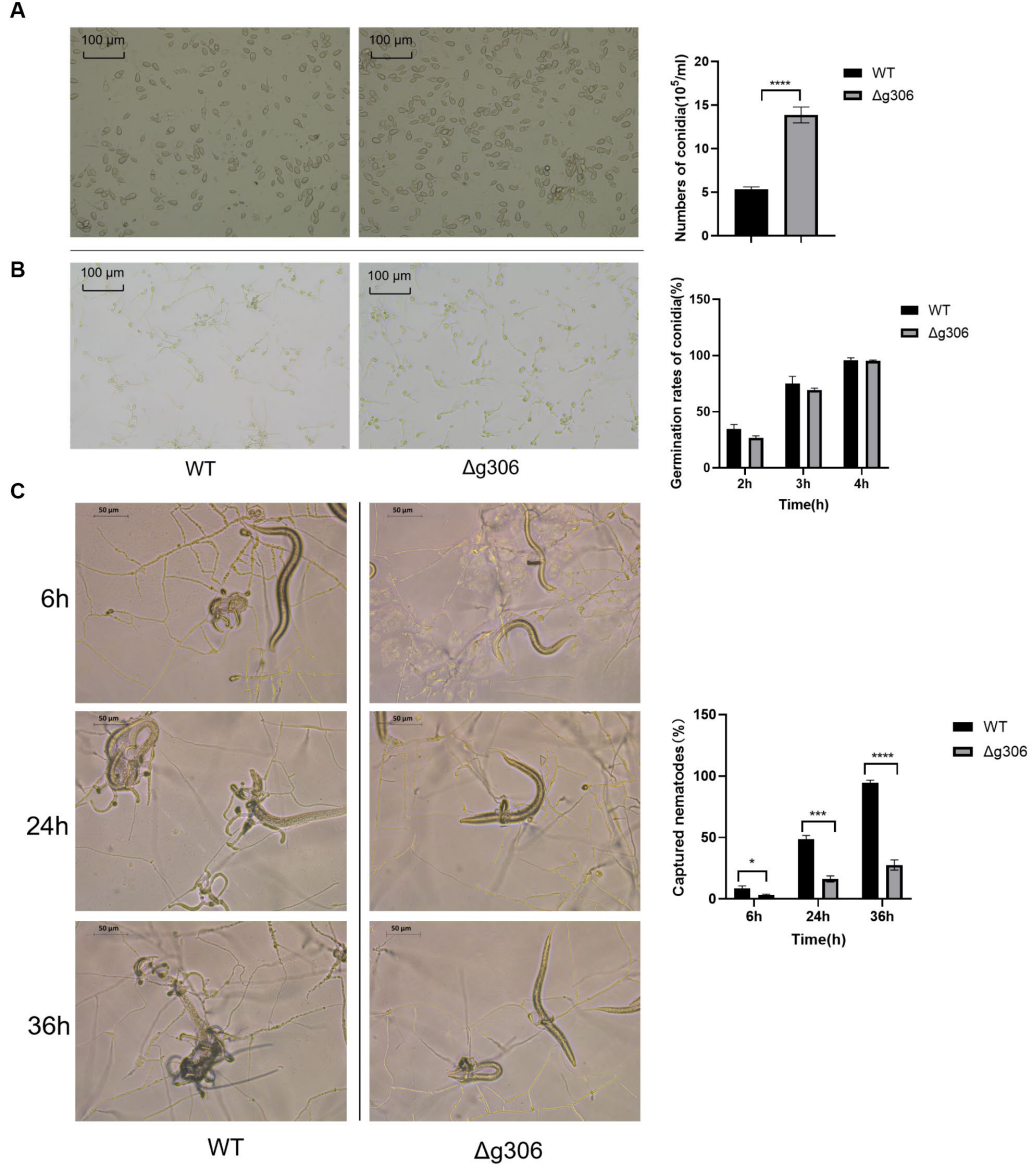
Effects of *g306* disruption on spore production, spore germination and nematode predation efficiency of *Arthrobotrys oligospora*. **(A)** Number of conidia produced by wildtype (WT) and Δ*g306* mutants on CMA plates (per mL of spore suspension). **(B)** Germination rates of WT and Δ*g306* mutants spores grown on water agar plates for 2, 3, and 4 h. **(C)** Comparison of *A. oligospora* WT and Δ*g306* mutants for trap formation and predatory nematodes.

Spores of the WT and Δ*g306* strains were collected after 10 days of incubation on CMA plates, and the spore count, using a hematocrit plate, showed a spore concentration of 13.9 × 10^5^/mL for the Δ*g306* strain, which was approximately 2.6 times that of the WT strain (5.3 × 10^5^/mL) (Figure 4A). The spore suspension was spread on water agar plate, and observed after 2 h, and the spore germination rates of the WT and Δ*g306* strains were measured. The WT spore germination rate reached 34.7%, whereas that of the Δ*g306* mutant strain was 26.8%, which was 22.8% lower than that of the WT strain. After 3 h, the WT spore germination rate reached 75.2%, whereas that of the Δ*g306* mutant strain was 69.4%, slightly lower than that of the WT strain. After 4 h, the conidia of the WT and Δ*g306* strains were fully germinated, with germination rates of 95.7 and 95.2%, respectively, showing no significant difference (Figure 4B). The germination rates of the mutant strains were lower than those of the WT strains only at an early stage.

### 3.4. *g306* disruption impaired the nematode predation efficiency of *Arthrobotrys oligospora*

Nematodes were added to water agar plates coated with WT and Δ*g306* strains. After 6 h, approximately 9% of the nematodes were captured on the WT plates, while only 3% of the nematodes were captured on the Δ*g306* plates, representing only approximately 1/3 of the number of nematodes captured by the WT strain (*p <* 0.001). After 24 h, the WT strain had formed a large number of typical predatory traps that existed in clusters, with approximately 47% of the nematodes being captured; however, the Δ*g306* strain formed only a single predatory trap, and the nematode capture rate was only 16%, which was 66% less than the WT strain. After 36 h, 95% of the nematodes were predated and digested by the WT strain, whereas the nematode predation rate of the Δ*g306* strain reached only 28%, which was 1/3 less than that of the WT strain (Figures 4C).

### 3.5. *g306* disruption results in global metabolic reprogramming

Based on the above phenotypic results, *g306* disruption has a significant effect on *A. oligospora* stress resistance, spore production, and nematode predation efficiency. Sequence analysis indicated that its encoded product is a multidomain protein with a wide substrate spectrum. Thus, the effects of *g306* disruption on growth, development, and nematode predation efficiency are most likely to be a global metabolic remodeling event. To reveal the characteristics of global metabolic changes, a non-targeted metabolomic analysis was performed in the WT and Δ*g306* strains.

**Figure 5:**
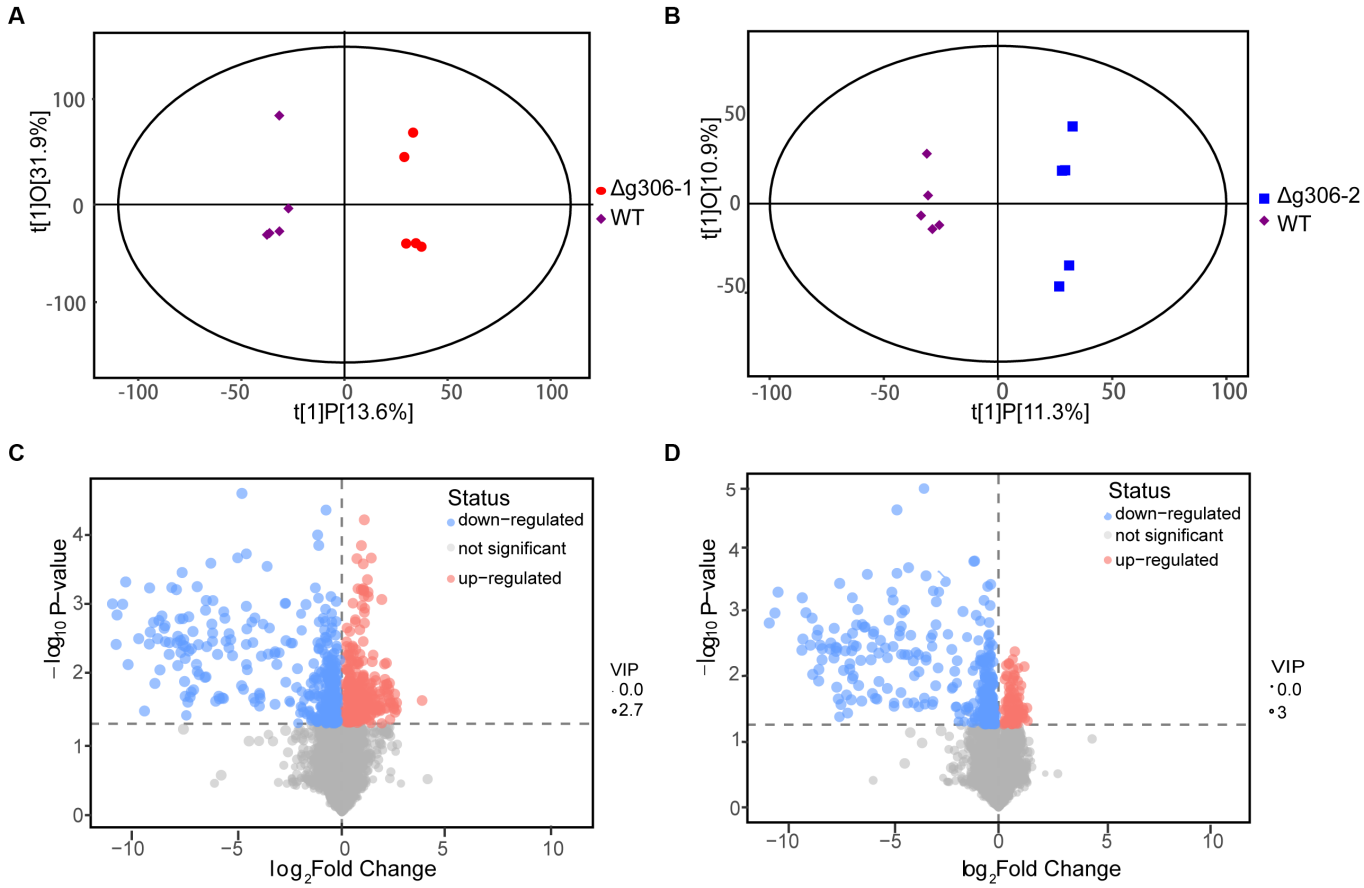
Metabolomics analysis. **(A)** Score scatter plot of the orthogonal partial least squares-discriminant analysis (OPLS-DA) model for group Δ*g306*-1 vs. wildtype (WT). **(B)** Score scatter plot of OPLS-DA model for group Δ*g306*-2 vs. WT. The horizontal coordinates show the differences between sample groups and the vertical coordinates show the differences within sample groups. **(C)** Volcano plot for group Δ*g306*-1 vs. WT. **(D)** Volcano plot for group Δ*g306*-2 vs. WT.

We used the orthogonal partial least squares-discriminant analysis (OPLS-DA) method to compare the Δ*g306*-1 and Δ*g306*-2 groups with the WT samples (Figures 5A,B). Scatter plots of the OPLS-DA model scores showed little within-group variation, indicating good within-group reproducibility. Some lateral distance was observed between the WT and knockout strain samples, indicating some variability between the metabolite species of the two groups.

To determine the metabolites that differed between the mutant and WT strain, we performed a differential metabolite screening (VIP > 1, *p* < 0.05). The results are presented as a volcano plot to visualize the overall distribution of the metabolite differences between the groups. Figures 5C,D show the differential metabolites between the Δ*g306*-1 group and the WT and those between the Δ*g306*-2 group and the WT, respectively. A total of 131 metabolites were altered in Δ*g306*-1 compared to those in the WT, of which 48 were significantly up-regulated and 83were significantly down-regulated. Of the 101 differential metabolites in Δ*g306*-2 compared to those in the WT, most were significantly down-regulated, with 19 being up-regulated and 82 being down-regulated (Supplementary material S2).

**Figure 6:**
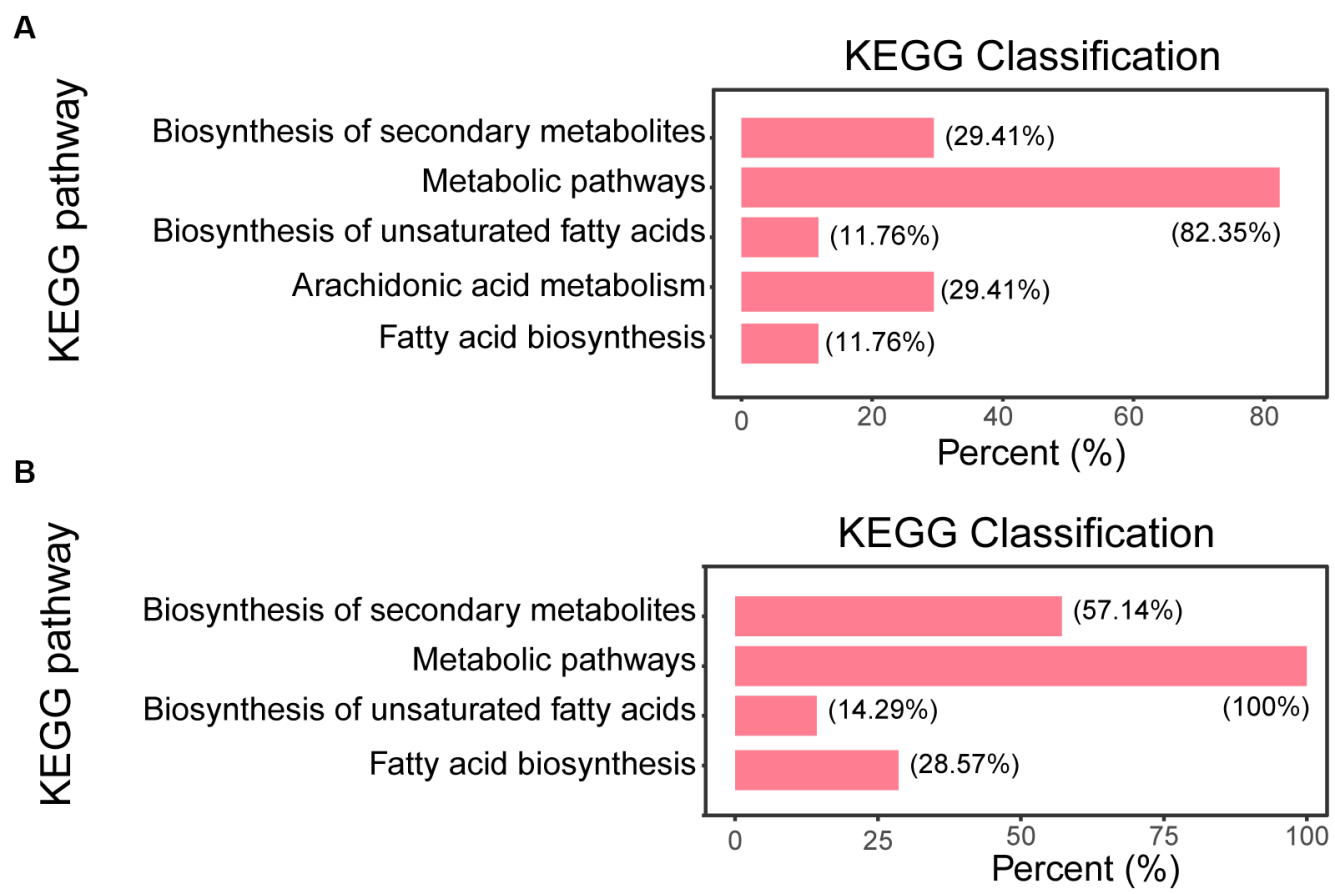
Pathway enrichment analysis. **(A)** Δ*g306*-1 vs. wildtype (WT); **(B)** Δg*306*-2 vs. WT. The horizontal coordinate indicates the number of differential metabolites annotated under a particular pathway, expressed as a percentage of the total number of differential metabolites annotated in all pathways. The vertical coordinate indicates the name of the enriched KEGG metabolic pathway.

The Kyoto Encyclopedia of Genes and Genomes (KEGG) enrichment analysis was based on the fungi database, we sorted out all the pathways of differential metabolite mapping of corresponding fungi species Zymoseptoria tritici (ztr), and used them as reference pathway library. The KEGG classification of the differential metabolites of the Δ*g306* group compared to those in the WT group is shown in Figure 6 (Supplementary material S2). The metabolites with significant differences between the Δ*g306*-1 group and the WT group were mainly concentrated in 5 metabolic pathways, including metabolic pathways, biosynthesis of unsaturated fatty acids, arachidonic acid metabolism and fatty acids biosynthesis. The metabolites that were significantly different between the Δ*g306*-2 and WT groups were similarly concentrated in 4 metabolic pathways, mainly including metabolic pathways, biosynthesis of unsaturated fatty acids, arachidonic acid metabolism and fatty acids biosynthesis, and these results were generally consistent with those of the Δ*g306*-1 and WT groups. Table 1 lists the differential metabolites in the Δ*g306*-1 group vs. WT and Δ*g306*-2 group vs. WT. Shared differential metabolites are mainly classified as fatty acyls, prenol lipids, steroids and steroid derivatives and amino acid derivatives etc. All these shared metabolites were down-regulated in the Δ*g306* strain compared to those in the WT strain. Thus, *g306* knockout affected the synthesis of these substances, subsequently leading to phenotypic changes associated with resistance and predatory trap formation.

**Table 1:** Significantly down-regulated metabolites in Δ*g306.*

Biomarkers (HMDB ID)	Metabolites Class	Δg306-1 vs. WT			Δg306-2 vs. WT		
		VIP	*p*	LOG_FOLDCHANGE	VIP	*p*	LOG_FOLDCHANGE
Stearic acid (HMDB0000827)	Fatty acyls	2.542462642	0.000121204	−1.230125298	2.093639776	0.020747592	−0.362272784
Glyceryl lactooleate (HMDB0032298)	Fatty acyls	2.509531696	0.000245641	−3.981875281	2.949043489	0.003214031	−9.28072389
Dodecanoic acid (HMDB0000638)	Fatty acyls	2.411076337	0.005926069	−0.741163481	2.88995902	0.0240365	−8.295404218
2-Hydroxystearic acid (HMDB0062549)	Fatty acyls	2.394629052	0.02093899	−1.454433326	2.533496801	0.019030141	−3.906664127
Alteichin (NA)	Lipopeptide	2.598336608	0.006740355	−3.464256644	2.005662411	0.032787534	−1.095752474
Byssochlamic acid (HMDB0034262)	Phenylpropanoids and polyketides	1.716397881	0.047674469	−0.235946499	2.787754443	0.019275152	−6.489329549
Griseorhodin A (NA)	Polyketide	2.69715972	0.002823557	−9.815799375	2.053538715	0.026537153	−0.313742564
Acidissiminin (HMDB0038619)	Prenol lipids	2.515219148	0.002920032	−5.919985763	1.988475305	0.038044035	−0.257731899
(24R)-5b,8b-Epidioxyergosta-6,22E-dien-3b-ol 3-glucoside (HMDB0037956)	steroid derivatives	2.699501421	0.004796555	−9.204169227	2.952348442	0.002262153	−10.30038072
7-Ketodeoxycholic acid (HMDB0000391)	Steroids and steroid derivatives	2.669770395	0.001348152	−8.162252182	2.840023047	0.002370031	−6.216078524
Calcidiol (HMDB0003550)	Steroids and steroid derivatives	2.159882584	0.044708183	−1.621560125	2.894730748	0.001359458	−7.473823647
Alanyl-Glutamic acid (NA)	Amino acid derivatives	2.535490686	0.000220832	−6.982023449	2.940392861	0.00479482	−9.291482145
3-Bromo-tyrosine (NA)	Amino acid derivatives	1.878134654	0.020200241	−0.286834364	2.792193436	0.007359892	−4.191350072
Phenylalanyl-Aspartate (HMDB0028991)	Carboxylic acids and derivatives	2.137387039	0.007166019	−0.24637113	2.175005449	0.00710879	−1.359479902
Aspartylphenylalanine (HMDB0000706)	Carboxylic acids and derivatives	1.813890786	0.025213891	−0.281614329	2.833174537	0.019800773	−6.402593757

## 4. Discussion

In the present study, we defined the sequence characteristics and identified the effects of *g306* on the cell metabolite profile cloud to obtain more information and determine the association between *g306* disruption and the multi-phenotypic changes in *A. oligospora*. Sequence and metabolomics analyZes demonstrated that *g306* is an A_NRPS (cd05930) and extended SDR_e1 (cd05235) domain-containing protein (Figure 1). The NRPS-like encoding gene *g306*, which encodes a protein, displayed a wide substrate spectrum. The encoded product of *g306* was annotated as a CP in the UniPort library. Metabolomics analysis indicated that *g306* disruption resulted in global and overview map changes. Specifically, the biosynthesis of secondary metabolites and lipid and amino acid derivatives metabolism were significantly altered (Figure 6), and some classes of metabolites, including fatty acyls, prenol lipids, steroids and steroid derivative, were identified as biomarkers after comparison of the metabolite profiles of the WT and Δ*g306* strains (Table 1). This was consistent with the sequence analysis results. CP domains are crucial components involved in the transfer of thiol ester-bound intermediates during the biosynthesis of primary and secondary metabolites such as fatty acids, polyketides, and non-ribosomal peptides (Witkowski et al., 1991; Byers and Gong, 2007; Qiao et al., 2007; Wattana-amorn et al., 2010). SDR_e1 consists of an SDR module of multidomain proteins identified as putative PKS, FASs, and NRPSs, among others, and catalyzes a wide range of activities, including the metabolism of steroids, cofactors, carbohydrates, lipids, aromatic compounds, and amino acids, and act in redox sensing (Persson et al., 2003; Kavanagh et al., 2008; Kramm et al., 2012; Borg et al., 2021). The differential metabolite screening results and biomarkers (Table 1) identified in our study, indicated that *g306* mainly regulates the biosynthesis of fatty acyls, prenol lipids, steroids and steroid derivatives, etc.

Fungi can produce diverse small-molecule secondary metabolites, mainly polyketides, non-ribosomal peptides, terpenoids, and alkaloids, and several families of enzymes, such as PKSs, NRPSs, and P450s, are commonly involved in the synthesis of these secondary metabolites (Keller et al., 2005). These secondary metabolites are biologically active mainly as inhibitors or toxins, e.g., metabolites produced by PKS–NRPS can act as toxins to kill infected cells or inhibit host defenses (Collemare et al., 2008). The formation of predation traps is a complex process involving many genes, proteins and metabolic pathways, considerable progress has been made in the understanding of the evolutionary and molecular mechanisms of fungal trap formation at the genomic, proteomic and transcriptional levels. When nematodes are induced to form traps, multiple fungal signaling pathways are activated and downstream genes associated with cell wall energy metabolism and biosynthesis, as well as adhesion proteins involved in trap formation, are regulated. Metabolomic studies have shown that the fungus produces a number of metabolites associated with the transition from the saprophytic to the pathogenic stage (Wang et al., 2018). Zhang et al. (2012) purified and characterized a novel set of metabolites, arthrosporols A-C, from *A. oligospora* that are involved in the regulation of fungal morphogenetic transformation. Compounds 1(A) and 3(C) played an important regulatory role in conidia formation and transformation of mycelia into a two-dimensional network. Regarding lipid and lipid-like molecular, some lipid and lipid-like molecules produced by *A. oligospora* play an important role in nematode predation. For example, linoleic acid was among the first nematocidal compounds isolated from *Arthrobotrys* NT fungi, and its quantity was positively correlated with the number of trapping devices formed by NT fungi during the predation phase (Stadler et al., 1993). In this study, lipid metabolic pathways were altered. Metabolomics data showed significant changes in the metabolism of amino acids. Amino acids have been reported to induce the formation of predatory traps in *A. oligospora* (Friman et al., 1985; Lu et al., 2023). Kuo et al. detected strain-specific small peptides in the predatory phase of *A. musiformis*, that are essential for the biochemical function of predation traps (Kuo et al., 2020). Proteomics and quantitative PCR analysis revealed significant up-regulation of many genes involved in early trap formation, including some genes involved in translation, amino acid metabolism, and carbohydrate metabolism (Yang et al., 2011). Non-ribosomal peptide synthesis systems, such as NRPSs, can synthesize non-ribosomal peptides using rare amino acids or fatty acids, such as, cyclosporine, daptomycin, bleomycin, and vancomycin, that have antibacterial, insecticidal, and anticancer activities (Liu et al., 2017). We compared the non-ribosomal peptide synthesis gene clusters with previously reported. Some of the most similar products, such as piperazine analogs, were identified. Morgan et al. reported in 2021 a unimodal NRPS, PVFC, encoded by the *Pseudomonas* virulence factor (PVF) gene cluster synthesizing pyrazinone, imidazole, pyrazines, etc. function as signaling molecules and virulence factors (Morgan et al., 2021). The chemical diversity of fungal metabolites plays an important role in the predatory phase of *A.oligospora*.

Overall, the functional characteristics of the NRPS-like encoding gene *g306* in *A. oligospora* were identified using gene knockout and metabolomics. We demonstrated that *g306* controls the biosynthesis of several classes of *A. oligospora* metabolites, including fatty acyls, prenol lipids, steroids and steroid derivatives, and amino acid derivatives, thereby regulating fungal growth, metabolism, and nematocidal efficiency. Our study also identified an association between NRPS-like genes and changes in the morphological features of NT fungi. The metabolite biomarkers we identified provide new strategies for the biocontrol of plant-parasitic nematodes based on NT fungi.

## Data availability statement

The original contributions presented in the study are included in the article/Supplementary material, further inquiries can be directed to the corresponding authors.

## Author contributions

TG performed the experiments and writing. HeL designed the project, investigation, supported metabolomics analysis and data interpretation, writing (review and editing), and funding acquisition. HuL supported metabolomics analysis and writing (review and editing). GZ: data interpretation and review and editing. YW: funding acquisition, supervision, and writing (review and editing). All authors contributed to the article and approved the submitted version.

## Funding

This research was funded by the National Natural Science Foundation of China [nos. 32200042, 31770066]; the Anhui Provincial Natural Science Foundation [no. 2208085QC67]; and the Natural Science Foundation of Anhui Higher Education Institutions of China [no. KJ2021A0058].

## Conflict of interest

The authors declare that the research was conducted in the absence of any commercial or financial relationships that could be construed as a potential conflict of interest.

## Publisher’s note

All claims expressed in this article are solely those of the authors and do not necessarily represent those of their affiliated organizations, or those of the publisher, the editors and the reviewers. Any product that may be evaluated in this article, or claim that may be made by its manufacturer, is not guaranteed or endorsed by the publisher.

## Supplementary material

The Supplementary material for this article can be found online at: https://www.frontiersin.org/articles/10.3389/fmicb.2023.1210288/full#supplementary-material
