# Supplementary material for: Function discovery of a non-ribosomal peptide synthetase-like encoding gene in the nematode-trapping fungus Arthrobotrys oligospora
Source: Front Microbiol. 2023 Jul 13;14:1210288. doi: 10.3389/fmicb.2023.1210288 (PMC10373296; doi:10.3389/fmicb.2023.1210288)
Supplement: Supplementary file 1 [file Table_1.docx]

Supplementary Material

**Function discovery of a non-ribosomal peptide synthetase-like encoding gene in the nematode-trapping fungus *Arthrobotrys oligospora***

**Tiantian Gu, Hengqian Lu*, Huiwen Liu, Guanghui Zhang，Yongzhong Wang***

***Corresponding author:** Yongzhong Wang **E-mail:** yzwang@ahu.edu.c

**Table S1. Primer sequences used in this study.**

| **Primer** | **Sequences (5’-3’)** |
| --- | --- |
| g306-3F | TAATCCTTCTTTAGAATAACACCCAGCTACTATAT AAGTATTGACTAATCTTG |
| g306-3R | AGTGAATTCGAGCTCGGTACCCGGGGATCCAAGT TAGCGTAATACAATAGGCTCAAAAACAG |
| g306-5F | AAGCTTGCATGCCTGCAGGTCGACTCTAGATAGA AACAACTCTGGAGGCCTGT |
| g306-5R | ATATCATCTTCTGAGTTTGAAGTTTGGGATCTTTT GAGGAGA |
| Hph-F | TGGGTGTTATTCTAAAGAAGGATTACCTCTAAAC AAGTGTACCTG |
| Hph-R | AAACTTCAAACTCAGAAGATGATATTGAAGGAG CATTTTTGGG |
| YZ-F | GCGAGAAATAGACCCATAGACAA |
| YZ-R | AAATCAAGCAGATGAGAAGAAGG |
